# RETRACTION: Risk Factors Contributing to Postoperative Surgical Site Infections in Patients Undergoing Ankle Fracture Fixation: A Systematic Review and Meta‐Analysis

**DOI:** 10.1111/iwj.70579

**Published:** 2025-04-18

## Abstract

**RETRACTION:** YinC. and SunL. , “Risk Factors Contributing to Postoperative Surgical Site Infections in Patients Undergoing Ankle Fracture Fixation: A Systematic Review and Meta‐Analysis,” International Wound Journal 21, no. 4 (2024): e14639, 10.1111/iwj.14639.38153200 PMC10961858

The above article, published online on 28 December 2023, in Wiley Online Library (http://onlinelibrary.wiley.com/), has been retracted by agreement between the journal Editor in Chief, Professor Keith Harding; and John Wiley & Sons Ltd. Following an investigation by the publisher, all parties have concluded that this article was accepted solely on the basis of a compromised peer review process. The editors have therefore decided to retract the article. The authors did not indicate their agreement with the retraction.

**RETRACTION:** C. Yin and L. Sun , “Risk Factors Contributing to Postoperative Surgical Site Infections in Patients Undergoing Ankle Fracture Fixation: A Systematic Review and Meta‐Analysis,” International Wound Journal 21, no. 4 (2024): e14639, 10.1111/iwj.14639.38153200 PMC10961858

The above article, published online on 28 December 2023, in Wiley Online Library (http://onlinelibrary.wiley.com/), has been retracted by agreement between the journal Editor in Chief, Professor Keith Harding; and John Wiley & Sons Ltd. Following an investigation by the publisher, all parties have concluded that this article was accepted solely on the basis of a compromised peer review process. The editors have therefore decided to retract the article. The authors did not indicate their agreement with the retraction.

